# Metathesis of Fatty Acid Ester Derivatives in 1,1-Dialkyl and 1,2,3-Trialkyl Imidazolium Type Ionic Liquids

**DOI:** 10.3390/ijms12063989

**Published:** 2011-06-14

**Authors:** Priya A. Thomas, Bassie B. Marvey, Eno E. Ebenso

**Affiliations:** 1 Department of Chemistry, North-West University (Mafikeng Campus), Private Bag X2046, Mmabatho 2735, South Africa; E-Mail: Priya.Thomas@nwu.ac.za; 2 Department of Chemistry, University of Limpopo, PO Box 197, Medunsa 0204, South Africa; E-Mail: Bassy.Marvey@ul.ac.za

**Keywords:** self-metathesis, methyl oleate, methyl ricinoleate, [bmim][X], [bdmim][X]

## Abstract

The self-metathesis of methyl oleate and methyl ricinoleate was carried out in the presence of ruthenium alkylidene catalysts **1**–**4** in [bmim] and [bdmim][*X*] type ionic liquids (RTILs) (*X* = PF_6_^−^, BF_4_^−^ and NTf_2_^−^) using the gas chromatographic technique. Best catalytic performance was obtained in [bdmim][X] type ionic liquids when compared with [bmim][*X*] type ionic liquids. Catalyst recycling studies were also carried out in the room temperature ionic liquids (RTILs) with catalysts **1**–**4** in order to explore their possible industrial application.

## 1. Introduction

Room temperature ionic liquids (RTILs) have been receiving considerable attention as innovative solvents for metal catalyzed organic reactions [1–6]. Most ionic liquids are based on ammonium, imidazolium, phosphonium, pyridinium, sulphonium, picolinium, pyrrolidinium, thiazolium, oxazolium, and pyrazolium cations. The important properties of ionic liquids, such as affinity to water and miscibility with other solvents, can be adjusted by the proper choice of alkyl substituents and counter anion. The most commonly used ionic liquids in olefin metathesis are 1,3-dialkylimidazolium and 1,2,3-trialkylimidazolium salts. Buijsman and co-workers [3] were the first group that reported the application of ionic liquids as a medium for olefin metathesis. They studied ring closing metathesis (RCM) of several substrates using Grubbs first and second generation catalysts in RTILs, and [bmim][PF_6_] was found to be the optimal solvent for the reactions. The activity and recyclability of Grubbs second generation catalyst in the self-cross-metathesis of some terminal olefins in RTILs has also been reported by Tang and co-workers [7]. Self-metathesis of 1-octene in 1,3-dialkylimidazolium and 1,2,3-trialkylimidazolium type ionic liquids have been reported by Williams and co-workers [8]. The results from the study showed that ionic liquids with shorter alkyl chain length enhanced the catalytic activity while the longer ones inhibited it. To overcome the problem associated with the leaching of products into the substrate, many charged catalyst systems have been employed [9–14].

**Scheme 1:**
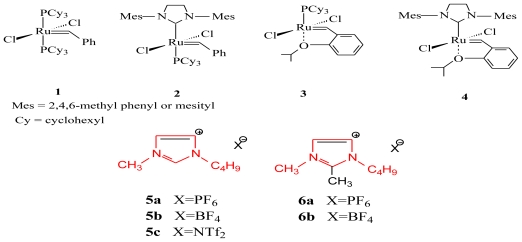
Ruthenium catalysts and imidazolium ionic salts.

The present work is an extension of our previous one which highlighted the self-metathesis reaction of methyl oleate in [bmim][*X*] type ionic liquids [15]. In this study, we report the self-metathesis of methyl oleate and methyl ricinoleate using the catalysts [**1**–**4**] in [bmim][*X*] and [bdmim][*X*] type ionic liquids (Scheme 1). The study also compares the metathesis activity in [bdmim] and [bmim] type ionic liquids. Catalyst recycling was also carried out in ionic liquids with the catalysts **1**–**4**.

## 2. Results and Discussion

### 2.1. Self-Metathesis of Methyl Oleate

**Figure 1:**
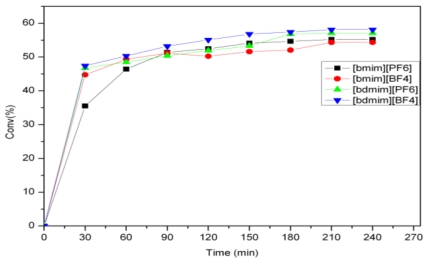
Activity of **3** for the self-metathesis of methyl oleate in different ILs.

**Figure 2:**
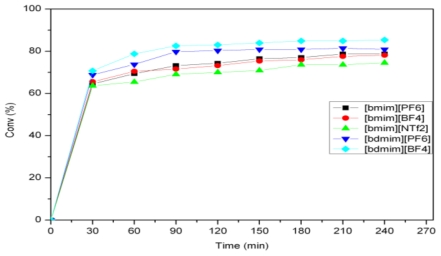
Activity of **4** for the self-metathesis of methyl oleate in different ILs.

The self-metathesis of methyl oleate was carried out in the presence of ruthenium alkylidene catalysts **1**–**4** at 40 °C. The Hoveyda-Grubbs catalysts **3** and **4** were evaluated in 1-butyl 3-methylimidazolium (bmim) and 1-butyl 2,3-dimethylimidazolium (bdmim) based ionic liquids with different anions (PF_6_^−^, BF_4_^−^ and NTf_2_^−^) (Scheme 1). Figure 1 shows the activity of **3** in [bmim] and [bdmim] type ionic liquids. For catalyst **3**, similar conversions were observed in [bmim] and [bdmim] type ionic liquids (ILs). As there was not much difference in the catalytic activity, the benefit of selecting a particular IL (among PF_6_^−^, BF_4_^−^ and NTf_2_^−^) was found to be of less importance. For catalyst **4**, the highest activity was found in [bdmim][BF_4_] with a conversion of 85% (Figure 2). Comparing the cations of the ionic liquids, the C2-methylated imidazolium cation [bdmim] led to a slightly better conversion. The benefit of using [bdmim] cation in the ethenolysis of methyl oleate has been previously reported by Thurier *et al.* [16].

To study the effect of temperature on metathesis activity, the reactions were conducted at temperatures 40 °C and 60 °C. The results are summarized in Table 1. An increase in substrate conversion was observed with increase in temperature. At both the temperatures, the highest substrate conversion was seen with catalyst **4**. Generally for all the runs, Hoveyda-Grubbs catalyst **4** exhibited good activity, although isomerization and formation of secondary metathesis products [17] seems to be a drawback. Hoveyda-Grubbs catalyst **3** and **4** showed good solubility in ionic liquid compared to Grubbs catalyst **1** and **2**. The reason for the high activity of the Hoveyda-Grubbs second generation catalyst can be attributed to the absence of phosphine ligand. It has been proved that phosphine ligand suppresses the catalyst activity in Ru-catalyzed olefin metathesis [18]. The presence of free phosphine in solution can inhibit coordination of olefins to the transition metal centre by re-association with the active Ru complex. With the non-phosphine Ru complex (catalyst **4**), activation occurs through the loss of O→Ru chelation. The styrenyl ether ligand completes less effectively with olefin substrates for Ru chelation [19]. [bdmim] type ILs can prevent the carbene formation of catalyst, which is believed to be formed by the deprotonation of imidazolium cation or from the oxidative addition of imidazolium to Ru centre [20].

### 2.2. Self-Metathesis of Methyl Ricinoleate

**Figure 3:**
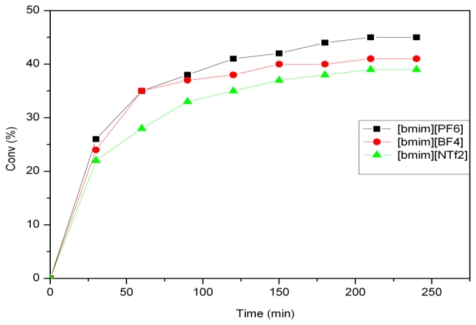
Activity of 1 for the self-metathesis of methyl ricinoleate in different ionic liquids.

**Figure 4:**
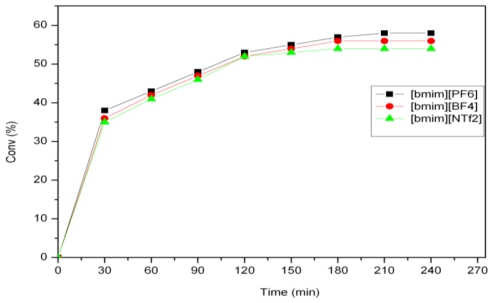
Activity of 2 for the self-metathesis of methyl ricinoleate in different ionic liquids.

**Scheme 2:**
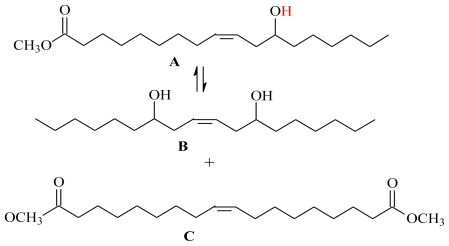
Primary metathesis products from self-metathesis of methyl ricinoleate.

The self-metathesis of methyl ricinoleate was carried out in the presence of catalysts **1** and **2** in [bmim][X] type ILs. Table 2 summarizes the activity of catalysts **1**–**4** for the self-metathesis of methyl ricinoleate. For catalyst **1**, the highest activity was shown in **5a** with a substrate conversion of 45% and the activity was found to be in the order PF_6_ > BF_4_ > NTf_2_ (Figure 3). For catalyst **2**, the same trend was followed, with the reaction in **5a** yielding the highest conversion of 58% (Figure 4). The primary metathesis products (PMP) obtained from the metathesis of methyl ricinoleate (**A**) were 9-octadecene- 7,12-diol (**B**) and dimethyl 9-octadecenedioate (**C**), as illustrated in Scheme 2 [21].

### 2.3. Catalyst Recycling in Ionic Liquids

As solvents, ionic liquids are more advantageous than conventional organic solvents, which make them recyclable and environmentally friendly. It has been reported that the ionic liquids could be reused for at least three runs [3,16,22,23]. In a typical experiment, metathesis reaction was carried out in ionic liquids, and, after extraction of reaction products with heptane (2 × 3 mL), the glass reactor was loaded with fresh methyl oleate and was introduced for a new run.

To study the recyclability, Ru catalysts **1**–**4**, in different ionic liquids, were subjected to consecutive runs (Table 3). Grubbs catalyst **1** proved to be stable in three consecutive runs, with the third run resulting in a decreased conversion. Grubbs catalyst **2** showed an increased conversion in the third cycle when compared with catalyst **1**. Comparing the Hoveyda-Grubbs catalysts **3** and **4**, the best result was obtained with catalyst **3**. The results prove that catalyst **3** can be reused for three runs without loss of activity. In spite of good activity in the first run, the second generation Hoveyda-Grubbs catalyst **4** showed low catalytic activity during the second run.

## 3. Experimental Section

### 3.1. Materials and Apparatus

1-Butyl-3-methylimidazolium hexafluorophosphate ([bmim][PF_6_]), 1-butyl-3-methylimidazolium tetrafluoroborate ([bmim][BF_4_]), 1-butyl-3-methylimidazolium bis(trifluoromethylsulphonyl)imide ([bmim][NTf_2_]), 1-butyl-2,3-dimethylimidazolium hexafluorophosphate ([bdmim][PF_6_]), 1-butyl-2,3- dimethylimidazolium tetrafluoroborate ([bdmim][BF_4_]), were all reagent grade chemicals from Sigma-Aldrich. Methyl oleate (≥99%) and methyl ricinoleate (>99%) were obtained from Sigma-Aldrich and were treated with activated alumina and stored under N_2_ atmosphere at a subzero temperature. Ethyl vinyl ether was purchased from Fluka. Nonadecane purchased from Fluka was used as the internal standard (**IS**). Ruthenium catalysts **1**–**4** were stored under N_2_ and used as purchased from Sigma-Aldrich. Chromatograms were obtained using Varian Star 3400 *CX* GC equipped with a DB-624 capillary column (J&W Scientific, 30 m × 0.53 mm) and a flame ionization detector (FID). The oven temperature was held at 200 °C and then increased to 270 °C at a rate of 20 °C min^−1^. The injector temperature was set at 270 °C and the detector temperature at 300 °C with N_2_ as carrier gas.

### 3.2. Metathesis Experiments

All the reactions were performed under a N_2_ atmosphere in a glass reactor fitted with a thermometer and a rubber septum. For the reaction in RTILs, 0.5 mL of the substrate was added to 1 mL of ionic liquid and stirred for 10 min to attain the reaction temperature. An internal standard (0.05 g) was added followed by the addition of 12.4 mg of catalyst. All the catalysts were soluble in ILs with the substrate forming a biphasic mixture with ILs. Samples were withdrawn by a syringe at regular time intervals for up to 4 hours. The reaction was terminated by immediately quenching with a few drops of ethyl vinyl ether [22]. The quenched sample was diluted with solvent and analyzed by GC.

## 4. Conclusions

For the self-metathesis of methyl oleate, Hoveyda-Grubbs catalyst **4** provided the best result with enhanced activity. Similar conversions were observed in all the three [bmim][*X*] type ionic liquids selected. However, when compared with [bdmim][*X*] type ionic liquids, higher activity was observed, which shows the superiority of [bdmim][*X*] type ionic liquids as reaction media. Furthermore, it was found that Hoveyda-Grubbs catalyst **3** could be reused for three consecutive runs without loss of activity. Overall, the use of ionic liquids in the metathesis reaction opens a route for the use of vegetable oil as a renewable source of raw materials for the chemical industry.

## Figures and Tables

**Table 1:** Activity of **3** and **4** for the self-metathesis of methyl oleate in [bmim] and [bdmim][*X*] type ionic liquids.

RTIL	Catalyst	Temperature (°C)	Conversion (%)	Selectivity a (%)
5a	1	40	57	99
	2		65	97
	3		55	99
	4		78	96
	1	60	59	99
	2		75	94
	3		57	98
	4		87	93
5b	1	40	58	99
	2		67	96
	3		56	99
	4		79	96
	1	60	61	99
	2		78	94
	3		59	97
	4		87	95
5c	1	40	57	99
	2		64	96
	3		54	99
	4		74	97
	1	60	60	99
	2		73	96
	3		59	99
	4		85	95
6a	3	40	57	99
	4		81	98
	3	60	60	97
	4		90	95
6b	3	40	58	99
	4		85	99
	3	60	60	97
	4	40	92	95

0.5 mL of MO (MO/Ru ratio = 100), 1 mL of IL, reaction time = 4 h;

a selectivity towards PMPs.

**Table 2:** Activity of **1** and **2** for the self-metathesis of methyl ricinoleate.

RTIL	Catalyst	Temperature (°C)	Conversion (%)	Selectivity a (%)
**5a**	**1**	60	45	99
	**2**		58	99
**5b**	**1**	60	41	99
	**2**		56	99
**5c**	**1**	60	38	99
	**2**	60	55	99

0.2 mL of MR (MR/Ru ratio = 100), 0.5 mL of IL, reaction time = 4 h;

a selectivity towards PMPs.

**Table 3:** Recycling of catalysts in [bmim][BF_4_] a and [bdmim][BF_4_] a,b.

Catalyst	Conversion (%)	Selectivity (%)
**1**	1st run: 57 (58)	99
	2nd run: 56 (56)	99
	3rd run: 42 (45)	98
**2**	1st run: 67 (72)	99
	2nd run:50 (60)	97
	3rd run:45 (57)	97
**3**	1st run: 56 (58)	99
	2nd run: 56 (58)	99
	3rd run: 49 (53)	99
	4th run: 22 (30)	99
**4**	1st run: 74 (85)	99
	2nd run: 40 (54)	95
	3rd run: 15 (23)	95

a All reactions were performed at 40 °C for 4 hours, substrate/Ru = 100;

b values in bracket are for [bdmim][BF_4_].
